# Concomitant Medication Use With Xiyanping Injection and the Risk of Suspected Allergic Reactions: A Nested Case–Control Study Based on China’s National Medical Insurance Database

**DOI:** 10.3389/fphar.2022.883407

**Published:** 2022-06-21

**Authors:** Xunliang Tong, Xiaochen Zhu, Chunping Wang, Yifan Zhou, Yingying Yan, Siyan Zhan, He Zhu, Sheng Han, Yinchu Cheng

**Affiliations:** ^1^ Department of Pulmonary and Critical Care Medicine, National Center of Gerontology, Beijing Hospital, Institute of Geriatric Medicine, Chinese Academy of Medical Sciences, Beijing, China; ^2^ International Research Center for Medicinal Administration, Peking University, Beijing, China; ^3^ Department of Pharmacy Administration and Clinical Pharmacy, School of Pharmaceutical Science, Peking University, Beijing, China; ^4^ Chongqing Bashu Secondary School, Chongqing, China; ^5^ Department of Pharmacy, Peking University Third Hospital, Beijing, China; ^6^ Department of Epidemiology and Biostatistics, School of Public Health, Peking University Health Science Center, Beijing, China

**Keywords:** Xiyanping injection, concomitant medication, allergic reaction, drug safety, nested case–control study

## Abstract

**Introduction:** Xiyanping injection (XYP), a type of Traditional Chinese Medicine, is widely used and often applied in combination with other medications in treating bronchitis, tonsillitis, and bacillary dysentery in China. In recent years, an elevated risk of allergic reactions has been observed following XYP, but whether concomitant medication use contributes to this risk is still unknown.

**Objective:** This study aims to investigate the association between the concomitant use of XYP and the 25 most frequently co-applied medications with suspected allergic reactions for China’s patients receiving XYP.

**Methods:** A nested case–control study was conducted using the sampling data from 2015 China’s Urban Employees Basic Medical Insurance and Urban Residents Basic Medical Insurance database. Four anti-allergic marker drugs were used to evaluate suspected allergic reactions. Univariate analyses and multivariable conditional logistic regression were conducted, and results were reported as odds ratios (ORs) with a 95% confidence interval (CI). Sensitivity analyses were performed on the expanded sample by including those prescribed with anti-allergic marker drugs on the same day as XYP and then stopped XYP on the next day.

**Results:** Out of 57,612 participants with XYP prescription, we obtained 949 matched case–control pairs. Multivariable conditional logistic regression revealed that seven concomitant medications including gentamicin [OR = 4.29; 95% CI (2.52, 7.30)], cefoperazone-sulbactam [OR = 4.26; 95% CI (1.40, 13.01)], lidocaine [OR = 2.76; 95% CI (1.79, 4.25)], aminophylline [OR = 1.73; 95% CI (1.05, 2.85)], ribavirin [OR = 1.54; 95% CI (1.13, 2.10)], potassium chloride [OR = 1.45; 95% CI (1.10, 1.91)], and vitamin C [OR = 1.32; 95% CI (1.03, 1.70)] were associated with increased risk, while cefathiamidine [OR = 0.29; 95% CI (0.16, 0.51)] was associated with reduced risk. Sensitivity analysis on 2,438 matched pairs revealed similar findings.

**Conclusion:** Increased risks for suspected allergic reactions were found for the concomitant use of XYP with seven medications. Our data suggest that gentamicin, cefoperazone-sulbactam, lidocaine, and ribavirin should be applied with precautions for patients receiving XYP, and further studies on drug interactions and allergy mechanisms are warranted.

## Introduction

Xiyanping injection (XYP) is a type of traditional Chinese medicine (TCM) with andrographolide total sulfonate as its main ingredient. Because of its antimicrobial, antivirus, and anti-inflammatory effects, immune regulation effect, and antipyretic effect (Zheng et al., 2020), XYP has been widely used in treating bronchitis, tonsillitis, bacillary dysentery, and other infectious diseases in China, with a particular heavy use in pediatrics in the treatment of hand, foot, and mouth disease and upper respiratory tract infections (Wang et al., 2014; Yin et al., 2015), and usually applied in combination therapy with other medications. The add-on effects of XYP in improving efficacy, relieving symptoms in a shorter time, and reducing the hospital length of stay compared to routine treatment have been proved by several studies, and it has recently been reported effective in improving the recovery of mild to moderate COVID-19 patients (Yin et al., 2015; Xu, 2016, 2017; Wang et al., 2018; Xiao et al., 2020; Zhang et al., 2021).

In recent years, concerns have been raised about XYP’s safety profile. Studies (Huang et al., 2017; Deng et al., 2018) showed that the most commonly reported adverse drug reactions (ADRs) of XYP were allergic reactions, mostly with clinical manifestations of rash and pruritus, but severe allergies such as anaphylactic shock have also been reported. Skin and subcutaneous tissue were the most affected organs which took up approximately 65–90% of ADRs of XYP (Ma et al., 2014; Li, 2015; Kong, 2016). The concomitant use of medications with XYP was very common in clinical practices according to large-scale multi-center studies (Wang et al., 2016; Deng et al., 2018), which reached as high as 95.7% in XYP’s ADR/ADE cases (Deng et al., 2018). However, the mechanism of allergic reactions caused by XYP remained unclear, and the safety impact of the concomitant use of medications with XYP has generally been under-explored. Therefore, our study aims to explore the association between the concomitant use of the most frequently prescribed medications with XYP and suspected allergic reactions.

## Materials and Methods

### Study Design and Data Source

A nested case–control study (NCCS) in a retrospective cohort was conducted using the 2015 survey sampling database of China’s Urban Employees’ Basic Medical Insurance (UE-BMI) and Urban Residents’ Basic Medical Insurance (UR-BMI) programs, which were both national insurance programs covering more than 500 million people in total by the end of 2015. The 2015 survey sampling data included 4.6 million participants consisting of both urban employees and residents, which covered 61 cities including 4 municipalities, 25 provincial capitals, and 32 prefecture-level cities. All inpatient and outpatient records were collected from the information system of local medical insurance administrative agencies. The database integrated demographic and clinical information of participants, as well as records on the prescriptions of medications, medical devices, and medical services. All data were de-identified to protect participant privacy. Detailed information on this database has been introduced and can be found in previous studies (Yang et al., 2017; Zhuo et al., 2019).

### Study Population

All participants who had XYP prescriptions from the 2015 UR-BMI and UE-BMI databases were included in the study cohort. Since prescription records were the only medication information that was available in the claims database, we assumed in this study that when participants were prescribed with given medication, they actually administered the medication.

The cases of this study were defined as participants with allergic reactions from the study cohort with prescriptions of four types of anti-allergic drugs (Table 1) following therapeutic guidelines of allergy (Yan and Guan, 2009; Muraro et al., 2014) and clinical expert consultations. This is due to the fact that allergic reactions were usually not actively recorded in claims databases and thus difficult to be directly identified. Thus alternatively, we used surrogate measurement, the medication treatment of allergy, as the signal of allergy events. Based on that, we first included all participants from the study cohort with anti-allergic drug prescriptions and imposed a 1-month “waiting time” as a washout period to obtain incident cases. Next, in considering that allergy events caused by XYP usually occurred within 24 h according to studies (Huang et al., 2017; Deng et al., 2018), we excluded participants with prescriptions of anti-allergic drugs before or more than 3 days after the prescription of XYP. Since medicine prescriptions in the database did not provide accurate “time” information, it is impossible to identify the order of XYP and marker drugs when their prescription dates were the same. So, we further excluded participants with anti-allergic drugs and XYP prescribed on the same day, and finally, got the case group for the primary analyses of this study.

**TABLE 1:** Anti-allergic marker drugs.

Drug type	Standard drug name
Antihistamine	Promethazine
Corticosteroid	Dexamethasone
Calcium gluconate	Calcium gluconate
Adrenaline	Adrenaline

For each case subject, one control subject was selected from the same study cohort through 1:1 matching on the propensity score using a greedy algorithm (Parsons, 2001). A logistic regression model was used to estimate propensity scores with four covariates: age, gender, type of hospital visits (inpatient/outpatient), and hospital level (tertiary hospital/secondary hospital/primary healthcare institution).

### Exposure Definition

The exposure of the study was the 25 most frequently prescribed concomitant medications of XYP. These medications were selected from all the medications prescribed for case subjects on the same day of XYP’s prescription except anti-allergic drugs. All the medications were included regardless of route of administration, dosage, and frequency. For both cases and controls, exposure to the given target concomitant medication was identified when the participant had the concomitant medication prescription, and the prescription date should be the same as the prescriptions of XYP. In addition, for case subjects, such prescriptions should be no later than their prescriptions of anti-allergic drugs. The 25 medications were amoxicillin-clavulanate, penicillin, cefuroxime, cefathiamidine, cefoperazone-sulbactam, ceftriaxone, ceftazidime, cefazolin, meropenem, amikacin, gentamicin, azithromycin, levofloxacin, moxifloxacin, vitamin B6, vitamin C, aminophylline, ambroxol, heparin, ribavirin, lidocaine, pantoprazole, sodium bicarbonate, bromhexine, and potassium chloride.

### Statistical Analysis

We first conducted univariate analyses on each of the 25 target concomitant medications and then multivariable conditional logistic regression analyses on all of the 25 medications together using backward variable selection. Odds ratios (ORs) and 95% confidence intervals (CIs) were estimated between these concomitant medications used with XYP and suspected allergic reactions. An α level of 0.05 (two-tailed) was considered statistically significant. All analyses were performed using SAS (version 9.4, SAS Institute Inc., Cary, NC, United States).

To test the robustness of the results, we expanded the case group in the primary analyses by including participants who had anti-allergic marker drugs and XYP prescribed on the same day and stopped using XYP the next day. The underlying assumption was that the termination of XYP on the next day was to stop the allergic reactions induced by the XYP administered earlier. With this additional criterion, more cases with suspected allergy events were included in our analysis. The matching process for controls and all statistical analyses in sensitivity analyses were the same as primary analyses. Subgroup analysis was conducted by age group and gender in the expanded population.

## Results

**FIGURE 1:**
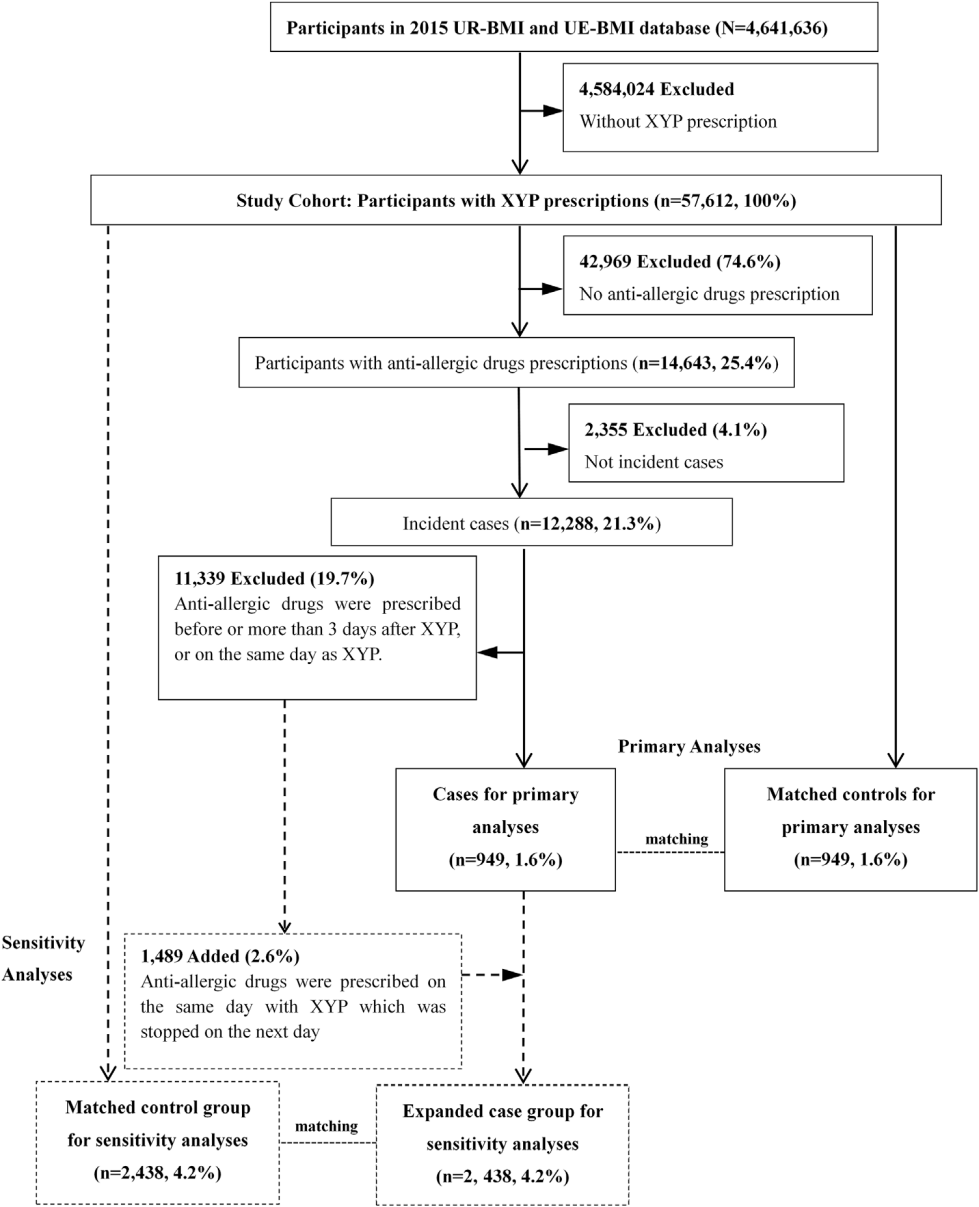
Flowchart of patient selection.

Out of the 4,641,636 participants in the 2015 UR-BMI and UE-BMI database, a total of 57,612 participants with XYP prescriptions were identified. After excluding 42,969 (74.6%) who had no anti-allergic marker drug prescription, we identified 12,288 (21.3%) as incident cases. Among incident cases, 11,339 (19.7%) were excluded, who had anti-allergic drugs prescribed before, more than 3 days after, or on the same day of XYP. We finally included 949 (7.7%) as cases for primary analyses, with 949 controls one-on-one matched from the same study cohort. An additional 1,489 cases were included with matched controls obtained from the study cohort for the sensitivity analysis. This expanded sample ended up with 2,438 cases matched with 2,438 controls (Figure 1).

### Characteristics of Cases and Controls

For the cases and controls of primary analyses, the average age ± standard deviation (SD) of the cases was 33.99 ± 29.56 years, 404 (42.57%) were children aged below 18 years; 504 (53.11%) were male, 231 (24.34%) received the first administration of XYP in the outpatient services while 718 (75.66%) received it during inpatient services. Secondary hospitals were the most frequently visited (*n* = 496, 52.27%), followed by primary health-care institution (*n* = 207, 21.81%), and tertiary hospitals (*n* = 246, 25.92%) were the least visited. As expected, the matched controls shared approximately the same distribution for baseline characteristics as the cases. Table 2 shows the baseline characteristics results.

**TABLE 2:** Baseline characteristics of patients with suspected allergic reactions and matched controls.

Characteristics	Cases (*N* = 949)	Controls (*N* = 949)
Age ( $\bar{x}$ ± SD)	33.99 ± 29.56	33.99 ± 29.57
Age group (n, %)		
<18 years	404 (42.57)	404 (42.57)
18–64 years	357 (37.62)	357 (37.62)
≥65 years	188 (19.81)	188 (19.81)
Gender (n, %)		
Male	504 (53.11)	505 (53.21)
Female	445 (46.89)	444 (46.79)
Type of hospital visits (*n*, %)		
Outpatient	231 (24.34)	231 (24.34)
Inpatient	718 (75.66)	718 (75.66)
Hospital level (n, %)		
Tertiary hospital	246 (25.92)	246 (25.92)
Secondary hospital	496 (52.27)	496 (52.27)
Primary health-care institution	207 (21.81)	207 (21.81)

SD, standard deviation.

### Univariate Analysis

Table 3 presents the univariate analysis results for 25 target concomitant medications in cases and controls, respectively. In summary, the combined use with a total of nine concomitant medications including vitamin C, potassium chloride, ribavirin, lidocaine, pantoprazole, gentamicin, aminophylline, amoxicillin-clavulanate, and cefoperazone-sulbactam was associated with the increased risk of allergic reactions. The combined use of 14 medications was found independent of the risk of allergic reactions, including ambroxol, vitamin B6, levofloxacin, azithromycin, penicillin, heparin, bromhexine, cefuroxime, sodium bicarbonate, cefazolin, ceftazidime, ceftriaxone, amikacin, and moxifloxacin. Moreover, the concomitant use of cefathiamidine was associated with decreased risk of allergic reactions.

**TABLE 3:** Univariate analyses results.

Concomitant medication	Cases with exposure (*N* = 949)	Controls with exposure (*N* = 949)	OR	95% CI	*p* Value
Ambroxol	274	242	1.19	0.97, 1.46	0.10
Vitamin C	268	203	1.50	1.20, 1.87	<0.01
Potassium chloride	209	159	1.43	1.13, 1.81	<0.01
Ribavirin	159	105	1.75	1.31, 2.34	<0.01
Vitamin B6	114	118	0.96	0.73, 1.26	0.78
Levofloxacin	97	75	1.34	0.97, 1.86	0.07
Lidocaine	93	38	2.83	1.87, 4.30	<0.01
Pantoprazole	90	67	1.41	1.00, 1.99	0.05
Azithromycin	86	75	1.16	0.84, 1.61	0.36
Gentamicin	84	20	4.56	2.74, 7.59	<0.01
Penicillin	77	85	0.90	0.66, 1.24	0.52
Heparin	72	64	1.15	0.80, 1.64	0.46
Bromhexine	65	74	0.87	0.62, 1.23	0.44
Cefuroxime	60	55	1.10	0.75, 1.62	0.62
Aminophylline	56	33	1.88	1.17, 3.03	0.01
Sodium bicarbonate	45	52	0.85	0.56, 1.30	0.85
Cefazolin	44	32	1.39	0.87, 2.20	0.16
Amoxicillin-clavulanate	43	24	1.83	1.10, 3.04	0.02
Ceftazidime	30	23	1.32	0.76, 2.29	0.33
Ceftriaxone	23	17	1.35	0.72, 2.53	0.34
Cefathiamidine	18	52	0.33	0.19, 0.58	<0.01
Cefoperazone-sulbactam	17	4	4.25	1.43, 12.63	<0.01
Meropenem	10	0	—	—	—
Amikacin	9	4	2.25	0.69, 7.30	0.07
Moxifloxacin	9	13	0.67	0.27, 1.63	0.37

OR, odds ratio; CI, confidence interval.

**FIGURE 2:**
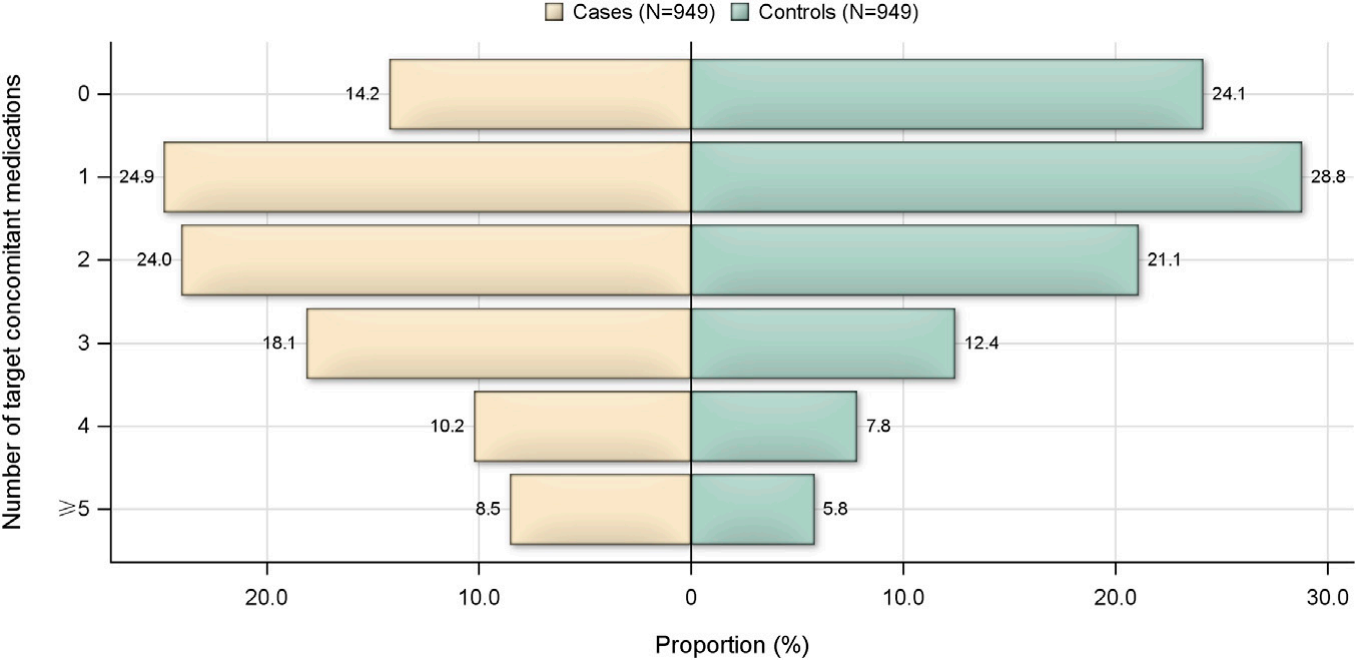
Number of target concomitant medications used by patients in the case and control groups.

The number of target concomitant medications used was also analyzed. The results showed the proportion of patients who used 0 pre-specified concomitant medication was much lower in the case group than that in the control group (14.2% vs. 24.1%), and the case group had a higher proportion of patients with multiple concomitant medications (Figure 2). Compared with no use of target concomitant medication, patients who used ≥1 type of concomitant medications had a 92% higher risk of developing allergic reactions (OR = 1.92; 95% CI, 1.52–2.43).

### Multivariable Conditional Logistic Regression Analysis

The results of multivariable conditional logistic regression on 25 target concomitant medications showed that the combined use of XYP with seven medications were associated with increased risk of allergic reactions, including gentamicin [OR = 4.29; 95% CI (2.52, 7.30)], cefoperazone-sulbactam [OR = 4.26; 95% CI (1.40, 13.01)], lidocaine [OR = 2.76; 95% CI (1.79, 4.25)], aminophylline [OR = 1.73; 95% CI (1.05, 2.85)], ribavirin [OR = 1.54; 95% CI (1.13, 2.10)], potassium chloride [OR = 1.45; 95% CI (1.10, 1.91)], and vitamin C [OR = 1.32; 95% CI (1.03, 1.70)], while the concomitant use with cefathiamidine [OR = 0.29; 95% CI (0.16, 0.51)] was the only one found associated with reduced risk of allergic reactions (Table 4).

**TABLE 4:** Multivariable conditional logistic regression analysis results.

Concomitant medication	OR	95% CI	*p* Value
Gentamicin	4.29	2.52, 7.30	<0.01
Cefoperazone-sulbactam	4.26	1.40, 13.01	0.01
Lidocaine	2.76	1.79, 4.25	<0.01
Aminophylline	1.73	1.05, 2.85	0.03
Ribavirin	1.54	1.13, 2.10	0.01
Potassium chloride	1.45	1.10, 1.91	0.01
Vitamin C	1.32	1.03, 1.70	0.03
Cefathiamidine	0.29	0.16, 0.51	<0.01

OR, odds ratio; CI, confidence interval.

### Sensitivity Analysis

After further including cases with anti-allergic marker drugs and XYP prescribed on the same day, and stopped using XYP on the next day, the sample for analysis was expanded to 2,438 cases matched with 2,438 controls, yielded a total of 4,876 for sensitivity analyses. Overall, sensitivity analysis results were consistent with the primary analyses. When concomitantly used with XYP, six medications remained associated with increased risk of allergic reactions, including gentamicin [OR = 6.34; 95% CI (4.19, 9.58)], lidocaine [OR = 4.48; 95% CI (3.33, 6.02)], aminophylline [OR = 2.53; 95% CI (1.76, 3.64)], ribavirin [OR = 1.76; 95% CI (1.44, 2.15)], vitamin C [OR = 1.49; 95% CI (1.26, 1.76)], and potassium chloride [OR = 1.39; 95% CI (1.14, 1.68)]. Cefathiamidine [OR = 0.63; 95% CI (0.44, 0.91)] remained associated with reduced risk (Supplementary Tables S1–S3).

### Subgroup Analysis

Results of subgroup analysis by age group and gender are shown in Supplementary Table S4. Generally, concomitant medications that may alter the risk of allergic reactions when combined with XYP injection are similar across the different age and gender subgroups and are consistent with the results of the primary analysis. Lidocaine, ribavirin, and gentamicin are associated with an increased risk of allergic reactions across all three age groups. Aminophylline, cefathiamidine, lidocaine, ribavirin, gentamicin, and vitamin C are significant in both male and female groups.

## Discussion

We performed a nested case–-control study using a national medical insurance database and found that 7 (gentamicin, lidocaine, aminophylline, ribavirin, vitamin C, potassium chloride, and cefoperazone-sulbactam) out of the 25 most commonly prescribed concomitant medications with XYP were associated with a higher risk of suspected allergic reactions, while the concomitant use with cefathiamidine was associated with decreased risk. Results from sensitivity analysis were consistent with the primary results, except for cefoperazone-sulbactam.

There have been *in vitro* and animal studies suggesting that andrographolide, the main ingredient of XYP injection, may have interactions with concomitant drugs through the cytochrome P450 (CYP450) enzyme and P-glycoprotein (P-gp) (Ye et al., 2011; Yu et al., 2021). A previous study has shown that XYP injection may alter the pharmacokinetics (PK) of lopinavir/ritonavir (Ye et al., 2021). CYP450/P-gp mainly affects the drug PK and leads to abnormal drug exposure, which may increase the risk of dose-dependent adverse events. However, in this study, the ADR of interest is acute allergic reactions that occurred within 3 days after drug exposure, which is usually not dose-dependent. Therefore, the allergic reactions are unlikely induced by drug interactions through the CYP450/P-gp pathway, but its impact on other dose-dependent adverse events still needs alert.

Among the seven concomitant medications that potentially increased the risk of allergic reactions, the influence of antibiotics, gentamicin, and cefoperazone sodium-sulbactam sodium were pronounced in primary analyses. Antibiotics were the most commonly used drug class in conjunction with XYP in the treatment of respiratory infections (Wang et al., 2016; Deng et al., 2018). The findings of our study are consistent with the established evidence that allergic reactions were common ADRs of antibiotics (Li et al., 2018).

Lidocaine, a widely used local anesthetic, was also found associated with a higher risk of allergic reactions when used concomitantly with XYP. Allergic reactions were listed as adverse reactions in the drug label information of lidocaine products. Notably, allergy caused by lidocaine in clinical uses has rarely been reported, but when it occurred, it was usually severe or even life-threatening (Bhole et al., 2012; Batinac et al., 2013; Jenerowicz et al., 2014; Kim et al., 2019). Mechanistically, the allergy was often thought to be the result of sensitivity to methylparaben, the preservative within the solution (Latronica et al., 1969; Speca et al., 2010; Grzanka et al., 2016; Hensley and Singer, 2018). In addition, our sensitivity results showed a substantial increase in risk after expanding the sample. These results indicate that type I hypersensitivity reaction, which usually occurs immediately, might be the major type of allergy associated with lidocaine. Our findings are supported by post-marketing adverse events reports showing that the majority of lidocaine’s ADRs occurred within 30 min (Wang, 2009; Shen et al., 2019).

Ribavirin is a broad-spectrum antiviral medication, and its concomitant use with XYP was also found in our study associated with an increased risk of allergic reactions. In the Chinese drug label, ribavirin injection is indicated to treat respiratory syncytial virus (RSV) induced pneumonia and bronchitis, but allergic reactions were not mentioned in it. However, allergic reactions associated with ribavirin have been reported in clinical studies on Chinese patients (Guo, 2014; Xu, 2016; Zhang, 2016; Yue, 2017; Zheng, 2019). While one study (Xu, 2016) observed fewer allergies to ribavirin + XYP combination therapy in treating respiratory infections compared to ribavirin monotherapy, several other studies (Guo, 2014; Zhang, 2016; Yue, 2017; Zheng, 2019) reported a higher incidence of allergic reactions for the concomitant use of ribavirin and XYP when using off-label in treating hand foot and mouth disease (HFMD), which was common in China’s clinical practices. As for the cause of allergy, an earlier study (Yang et al., 2013) on XYP’s compatibility with other medications showed that a great increase of subvisible particles was found for XYP and ribavirin injection, which exceeded the standard amount specified in Chinese Pharmacopoeia. Subvisible particles were one of the major causes of ADRs of TCM injections (Peng et al., 2018; Liu et al., 2019), which might account for the increased risk of allergic reactions. However, a clear mechanistic understanding of the concomitant use of ribavirin and XYP needs further research.

Aminophylline is the combination of theophylline and ethylenediamine (EDA). Aminophylline has been a frequently used bronchodilator in China and it was one of the most commonly used respiratory medications among all concomitant medications of XYP (Deng et al., 2018). The superior effects of aminophylline + XYP combination therapy compared to conventional therapy in relieving symptoms of airway obstruction and improving lung function have been proved by published studies (Ji, 2012; Wang et al., 2018). In our study, the concomitant use of XYP and aminophylline was found associated with an increased risk of allergic reactions. However, since the evidence of allergic reactions related to aminophylline was not well established, such finding in our study demands further evaluation.

The last two concomitant medications with increased risk of suspected allergic reactions were potassium chloride and vitamin C, which were both frequently used medications in supplementary or supporting treatment of XYP’s indications. Potassium chloride is mainly used to maintain the electrolyte balance of patients. Allergic reactions induced by this medication are rare, and the allergy mechanism is unknown (Tu and Peng, 2008). Vitamin C is one of the most commonly used concomitant medications of XYP (Wang et al., 2016; Deng et al., 2018), and it is usually used in combination with XYP to help enhance the immune function of patients. According to some previous studies (Wang and Xie, 2012; Deng et al., 2018), its concomitant use with XYP might be associated with the increase in ADR incidence, but no details were provided and the allergy mechanism was unclear.

According to a few studies (Wang, 2009; Ji, 2012), dexamethasone, one of the marker anti-allergic drugs, could be applied in combination with lidocaine or aminophylline to treat indications of XYP like asthma or asthmatic bronchitis. Under such conditions, using the prescription records of dexamethasone to signal the occurrence of allergic reactions would bring in confounding factors. However, the evidence of the therapeutic effectiveness of such combination therapy has not been well established, and such treatment was not common in clinical practices. Even though, with full awareness of the potential confounding, we have interpreted our results with great caution.

The major strength of this study is the use of real-world data at the national level, sourced from a large national medical insurance database that is widely covered and well represents the urban population of the country. The nested case–control study design and greedy matching on propensity scores made the cases and controls comparable and well balanced. Since both case and control subjects were from the same study cohort, some potential confounding bias could be minimized.

This study also has some limitations. First, we used surrogate measurement for allergic reactions due to a lack of direct ADR information in the claims database. Although many previous studies based on claims data have used medication prescription or healthcare resource utilization to define the underlying cases and assess the severity of illness (Petri et al., 1988; Bernatsky et al., 2011; Jacob et al., 2017; Arnaud et al., 2018; Ortsäter et al., 2021), there are still chances of misclassification. To minimize the false classification of cases and controls, several efforts were taken in the study design: marker drugs were carefully selected with consultation of clinical experts and pharmacists, a 1-month washout period was set to obtain incident cases, and a fairly conservative algorithm was applied in the identification of cases. Second, the prescription time was only accurate to date, making it impossible to determine the treatment sequence for patients who use XYP injection and marker drugs on the same day (i.e., same-day patients). To reduce potential bias, we adopted a conservative definition of case subjects in the primary analysis by excluding the same-day patients, and cautiously expanded the case group in the sensitivity analysis by further including same-day patients who stopped XYP on the next day. The results were consistent and supported the robustness of the analyses.

In this study, we only focused on assessing the risk of allergic reactions brought by the concomitant use of medications with XYP, compared to not using target concomitant medications. Additional comparisons between participants using XYP + concomitant medication and those not using XYP would be of value. For medications that have allergic reactions as their own adverse effect, this evidence could fill the gap in estimating the magnitude of risks induced by the combined use of XYP and concomitant medication. Furthermore, to better understand the allergy mechanisms associated with these medications and XYP, further investigations on the drug interaction effects would be indispensable.

## Conclusion

Increased risks for suspected allergic reactions were found for the concomitant use of XYP with seven medications. Gentamicin, cefoperazone-sulbactam, lidocaine, and ribavirin should be applied with precautions for patients using XYP. The suggested associations in our study for aminophylline, potassium chloride, and vitamin C demand further investigation. Future studies on drug interactions and allergy mechanisms are warranted to better understand the safety impact and provide references in the revision of XYP’s drug label information with additional safety notices.

## Data Availability

The data analyzed in this study are subject to the following licenses/restrictions: Extracted data that support the findings of this study are available from the Chinese Health Insurance Research Association but restrictions apply to the availability of these data, which were used under license for the current study and thus are not publicly available. Data are however available from the authors upon reasonable request and with permission of the Chinese Health Insurance Research Association. Requests to access these datasets should be directed to cyc_pku@bjmu.edu.cn
