# Supplementary material for: Concomitant Medication Use With Xiyanping Injection and the Risk of Suspected Allergic Reactions: A Nested Case–Control Study Based on China’s National Medical Insurance Database
Source: Front Pharmacol. 2022 Jun 21;13:883407. doi: 10.3389/fphar.2022.883407 (PMC9253428; doi:10.3389/fphar.2022.883407)
Supplement: Supplementary file 1 [file DataSheet1.docx]

Supplementary Material

# Supplementary Tables

**Supplementary Table 1.** Sensitivity analysis results: baseline characteristics of patients with suspected allergic reactions and matched controls

| **Characteristics** | **Cases**  **(N=2,438)** | **Controls**  **(N =2,438)** |
| --- | --- | --- |
| Age($\bar{x}$± SD) | 36.06±27.62 | 36.06±27.62 |
| Age group (n,%) |  |  |
| <18 years | 847 (34.74) | 847 (34.74) |
| 18-64 years | 1136 (46.60) | 1136 (46.60) |
| ≥65 years | 455 (18.66) | 455 (18.66) |
| Gender (n,%) | | |
| Male | 1335 (54.76) | 1335 (54.76) |
| Female | 1103 (45.24) | 1103 (45.24) |
| Type of Hospital Visits (n,%) | | |
| Outpatient Service | 1067 (43.77) | 1067 (43.77) |
| Hospitalization | 1371 (56.23) | 1371 (56.23) |
| Hospital Level (n,%) | | |
| Tertiary hospital | 808 (33.14) | 808 (33.14) |
| Secondary hospital | 1003 (41.14) | 1003 (41.14) |
| Primary health-care institution | 627 (25.72) | 627 (25.72) |

Abbreviations: SD, Standard Deviation.

**Supplementary Table 2.** Sensitivity analysis results: univariate analyses results

| **Concomitant Medication** | **Cases with Exposure**  **(N=2,438)** | **Controls** **with Exposure**  **(N=2,438)** | **OR** | **95%CI** | **P value** |
| --- | --- | --- | --- | --- | --- |
| Vitamin C | 633 | 407 | 1.81 | 1.56, 2.09 | <0.01 |
| Ambroxol | 631 | 559 | 1.18 | 1.03, 1.35 | 0.01 |
| Potassium Chloride | 469 | 304 | 1.73 | 1.47, 2.03 | <0.01 |
| Ribavirin | 378 | 234 | 1.87 | 1.55, 2.26 | <0.01 |
| Lidocaine | 310 | 81 | 4.82 | 3.65, 6.36 | <0.01 |
| Vitamin B6 | 294 | 238 | 1.27 | 1.06, 1.52 | 0.01 |
| Levofloxacin | 281 | 241 | 1.21 | 1.00, 1.47 | 0.05 |
| Pantoprazole | 260 | 153 | 1.91 | 1.53, 2.39 | <0.01 |
| Heparin | 238 | 114 | 2.32 | 1.82, 2.95 | <0.01 |
| Gentamicin | 203 | 32 | 6.90 | 4.67, 10.18 | <0.01 |
| Azithromycin | 196 | 198 | 0.99 | 0.80, 1.22 | 0.92 |
| Penicillin | 179 | 160 | 1.13 | 0.90, 1.41 | 0.28 |
| Bromhexine | 153 | 130 | 1.20 | 0.94, 1.54 | 0.15 |
| Cefuroxime | 146 | 142 | 1.03 | 0.81, 1.31 | 0.81 |
| Aminophylline | 128 | 60 | 2.36 | 1.70, 3.28 | <0.01 |
| Sodium Bicarbonate | 118 | 90 | 1.34 | 1.01, 1.79 | 0.04 |
| Cefazolin | 99 | 67 | 1.49 | 1.09, 2.04 | 0.01 |
| Amoxicillin and Clavulanate | 97 | 64 | 1.57 | 1.13, 2.17 | <0.01 |
| Ceftazidime | 82 | 59 | 1.40 | 1.00, 1.96 | 0.05 |
| Cefathiamidine | 71 | 90 | 0. 77 | 0.56, 1.07 | 0.12 |
| Ceftriaxone | 63 | 44 | 1.43 | 0.97, 2.10 | 0.07 |
| Cefperazone-Sulbactam | 40 | 15 | 2.66 | 1.47, 4.82 | <0.01 |
| Moxifloxacin | 40 | 32 | 1.28 | 0.79, 2.07 | 0.33 |
| Meropenem | 33 | 7 | 4.71 | 2.09, 10.66 | <0.01 |
| Amikacin | 20 | 10 | 2.00 | 0.94, 4.27 | 0.07 |

Abbreviations: OR, Odds ratio; CI, Confidence interval.

**Supplementary Table 3.** Sensitivity analysis results: multivariable conditional logistic regression analysis results

| **Concomitant Medication** | **OR** | **95% CI** | **P value** |
| --- | --- | --- | --- |
| Gentamicin* | 6.34 | 4.19, 9.58 | <0.01 |
| Lidocain* | 4.48 | 3.33, 6.02 | <0.01 |
| Meropenem | 2.54 | 1.05, 6.17 | 0.04 |
| Aminophylline* | 2.53 | 1.76, 3.64 | <0.01 |
| Ribavirin* | 1.76 | 1.44, 2.15 | <0.01 |
| Heparin | 1.60 | 1.23, 2.10 | <0.01 |
| Pantoprazole | 1.57 | 1.21, 2.03 | <0.01 |
| Amoxicillin-Clavulanate | 1.56 | 1.10, 2.21 | 0.01 |
| Vitamin C* | 1.49 | 1.26, 1.76 | <0.01 |
| Potassium chloride* | 1.39 | 1.14, 1.68 | <0.01 |
| Cefathiamidine* | 0.63 | 0.44, 0.91 | 0.01 |

Abbreviations: OR, Odds ratio; CI, Confidence interval; * refers to results which were also significant in primary analyses.

**Supplementary Table 4.** Subgroup analysis: multivariable conditional logistic regression analysis results

| **Subgroups and Concomitant Medications** | **OR** | **95% CI** | **P value** |
| --- | --- | --- | --- |
| **Age** |  |  |  |
| **<18 years (n=1694)** |  |  |  |
| Aminophylline* | 2.44 | 1.36-4.35 | <0.01 |
| Amoxicillin-Clavulanate | 1.98 | 1.25-3.13 | <0.01 |
| Cefathiamidine* | 0.52 | 0.33-0.82 | <0.01 |
| Cefoperazone-Sulbactam* | 3.75 | 1.21-11.68 | 0.02 |
| Ceftazidime | 1.77 | 1.04-3.03 | 0.04 |
| Gentamicin* | 4.56 | 2.10-9.86 | <0.01 |
| Lidocain* | 7.51 | 2.16-26.16 | <0.01 |
| Ribavirin* | 1.74 | 1.35-2.26 | <0.01 |
| **18~64 years (n=2272)** |  |  |  |
| Bromhexine | 2.11 | 1.23-3.50 | <0.01 |
| Gentamicin* | 5.49 | 3.21-9.38 | <0.01 |
| Heparin | 2.47 | 1.40-4.37 | <0.01 |
| Lidocain* | 4.40 | 3.06-6.34 | <0.01 |
| Pantoprazole | 1.57 | 1.11-2.21 | 0.01 |
| Penicillin | 1.97 | 1.08-3.61 | 0.03 |
| Ribavirin* | 1.59 | 1.12-2.26 | 0.01 |
| Vitamin C* | 1.80 | 1.38-2.35 | <0.01 |
| **≥65 years (n=910)** |  |  |  |
| Ambroxol | 1.49 | 1.06-2.10 | 0.02 |
| Aminophylline* | 2.11 | 1.23-3.50 | <0.01 |
| Cefathiamidine* | 0.19 | 0.04-0.97 | 0.05 |
| Gentamicin* | 10.42 | 2.94-36.94 | <0.01 |
| Heparin | 3.09 | 1.69-5.67 | <0.01 |
| Lidocain* | 4.60 | 2.43-8.67 | <0.01 |
| Ribavirin* | 1.59 | 1.12-2.26 | <0.01 |
| Sodium Bicarbonate | 6.94 | 1.90-25.35 | <0.01 |
| Vitamin C* | 3.40 | 2.12-5.44 | <0.01 |
| **Gender** |  |  |  |
| **Male (n=2670)** |  |  |  |
| Amoxicillin-Clavulanate | 1.58 | 1.01-2.47 | 0.05 |
| Aminophylline* | 2.53 | 1.55-4.11 | <0.01 |
| Cefathiamidine* | 0.51 | 0.29-0.88 | 0.02 |
| Cefoperazone-Sulbactam* | 2.72 | 1.22-6.09 | 0.02 |
| Cefazolin | 1.59 | 1.02-2.50 | 0.04 |
| Gentamicin* | 5.27 | 3.07-9.04 | <0.01 |
| Heparin | 1.70 | 1.20-2.39 | <0.01 |
| Lidocain* | 3.87 | 2.66-5.63 | <0.01 |
| Pantoprazole | 1.57 | 1.09-2.27 | 0.02 |
| Ribavirin* | 1.58 | 1.22-2.05 | <0.01 |
| Sodium Bicarbonate | 2.72 | 1.22-6.09 | 0.02 |
| Vitamin C* | 1.42 | 1.15-1.75 | <0.01 |
| **Female (n=2206)** |  |  |  |
| Amikacin | 5.00 | 1.02-24.64 | 0.05 |
| Aminophylline* | 2.33 | 1.34-4.06 | <0.01 |
| Cefathiamidine* | 0.55 | 0.32-0.96 | 0.04 |
| Gentamicin* | 8.81 | 4.52-17.18 | <0.01 |
| Heparin | 1.72 | 1.11-2.67 | 0.02 |
| Lidocain* | 5.69 | 3.47-9.32 | <0.01 |
| Pantoprazole | 1.67 | 1.15-2.41 | 0.01 |
| Potassium Chloride* | 1.41 | 1.03-1.94 | 0.03 |
| Ribavirin* | 2.07 | 1.49-2.88 | <0.01 |
| Vitamin C* | 1.92 | 1.46-2.53 | <0.01 |
| Vitamin B6 | 0.69 | 0.49-0.98 | 0.04 |

Abbreviations: OR, Odds ratio; CI, Confidence interval; * refers to results which were also significant in primary analysis.
